# Supplementary figures and images for: Assessment of distinct effects of Parinari curatellifolia Planch.ex Benth Ethanolic leaf extract on glucose transport in different cell types
Source: PeerJ. 2025 Nov 10;13:e20269. doi: 10.7717/peerj.20269 (PMC12614100; doi:10.7717/peerj.20269)

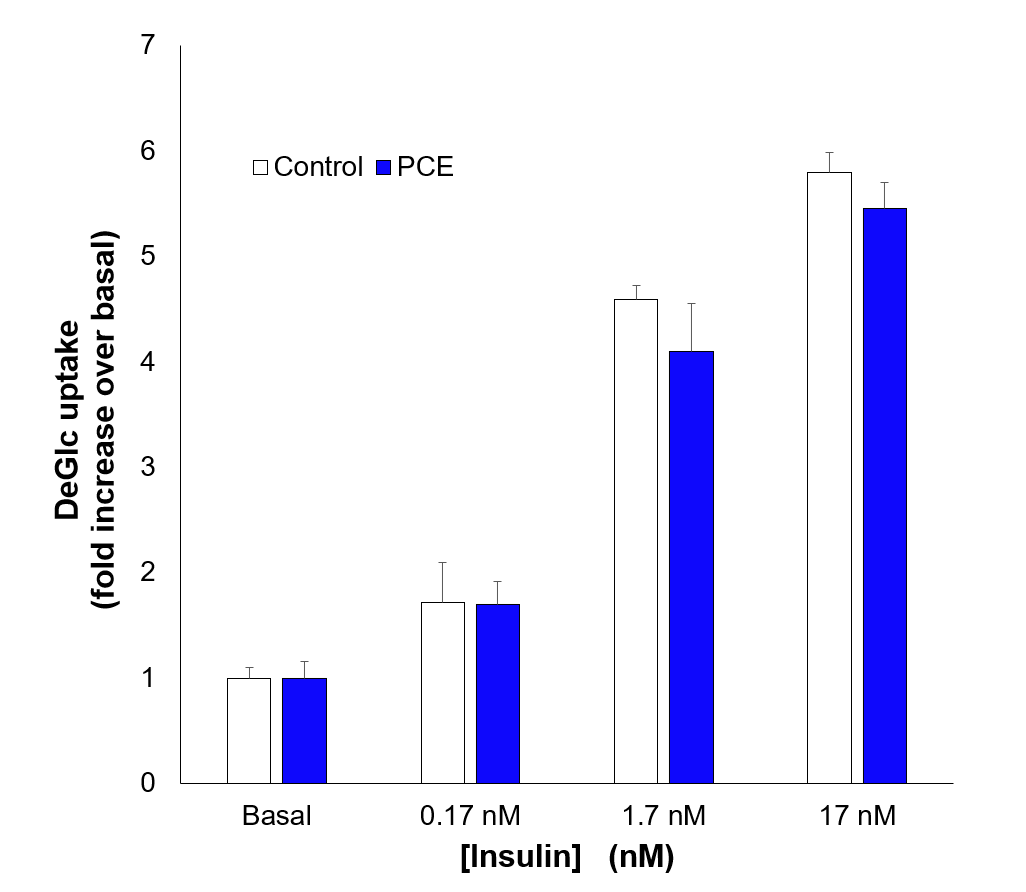

Supplement: Supplemental Information 1 — Using 3T3-L1 adipocytes as a model of adipocytes, we observed that 2h administration of PCE (100 µg/mL) had no effect on either insulin-independent or insulin-stimulated glucose transport. Shown is a representative experiment in which insulin was added at the concentration shown for 30 minutes after addition of PCE. Each bar is the average of triplicate technical replicates at each condition. Similar data was observed in three independent replicates of his type. [file peerj-13-20269-s001.png]

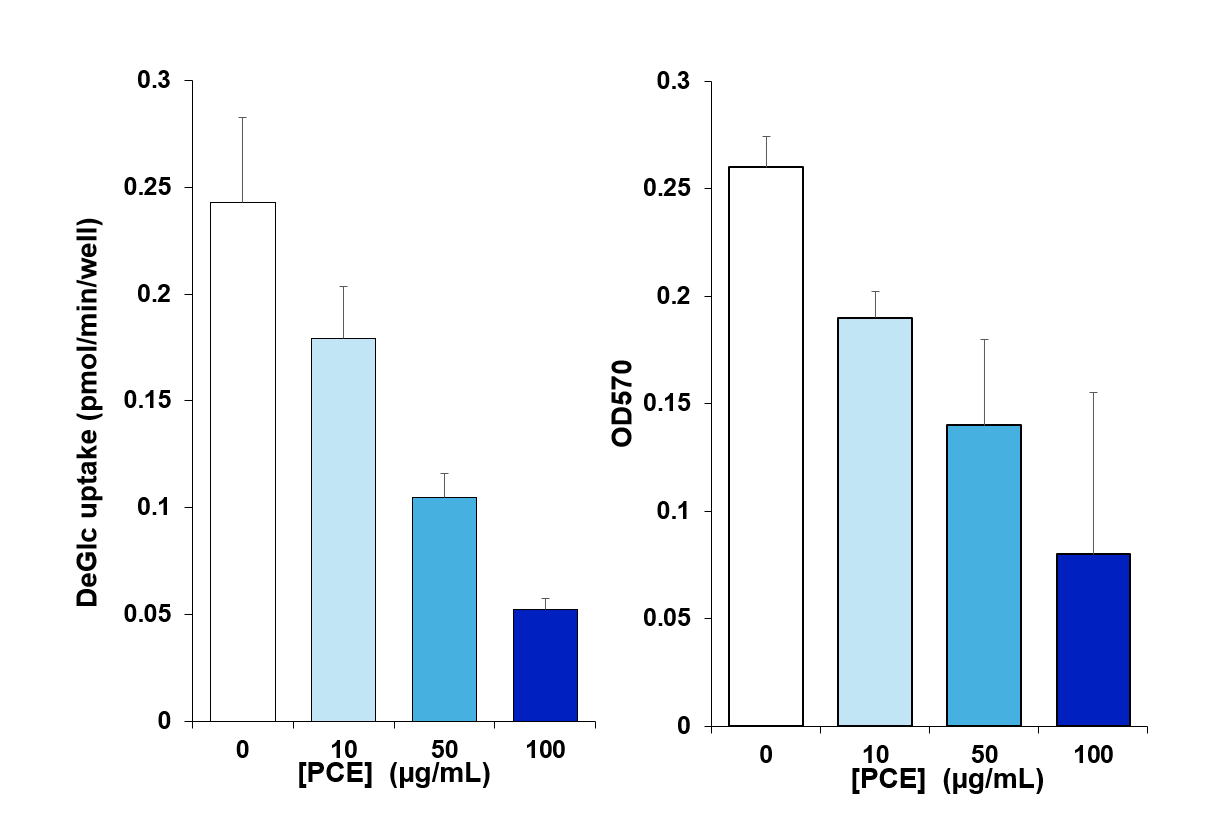

Supplement: Supplemental Information 2 — (A) 24h treatment with the indicated concentration of PCE significantly inhibited basal (unstimulated) deGlc uptake in H4IIE cells. Shown is a representative experiment in which each bar is the average of triplicate technical replicates. (B) Shows MTT assay data from the same cell plating, in which a clear reduction in cell viability is evident. Similar data was observed in three independent replicates of his type. [file peerj-13-20269-s002.png]

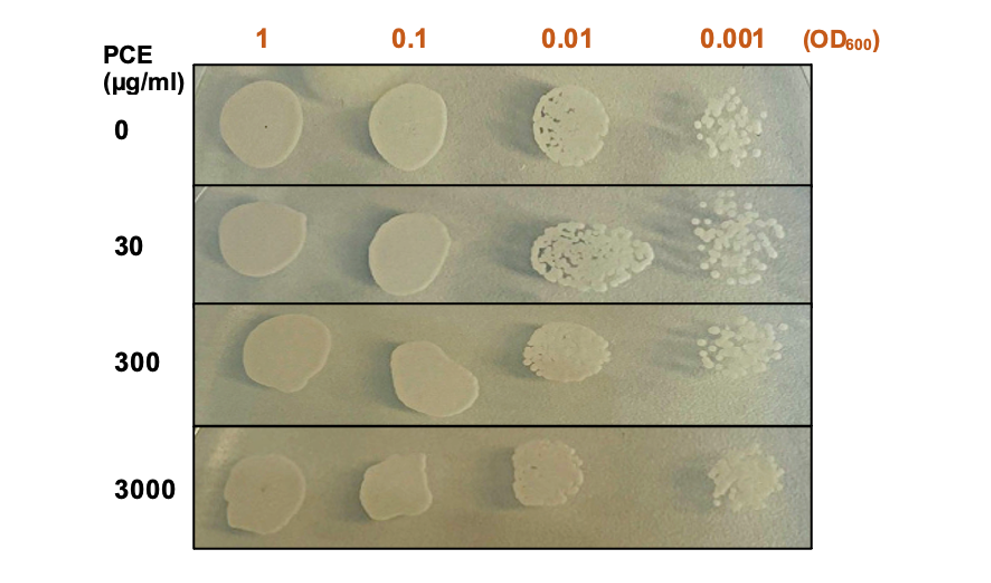

Supplement: Supplemental Information 3 — Wild type S. pombe cells were subjected to 10-fold serial dilution (1, 0.1, 0.01 and 0.001 OD600 measurement). Diluted cells were spotted onto PCE coated solid YE media plates at 0, 30, 300, & 3,000 µg/ml. Cell were grown at 30° C for 72 hours. Data from a representative experiment is shown, replicated three times. [file peerj-13-20269-s003.png]
