# Supplementary material for: Assessment of distinct effects of Parinari curatellifolia Planch.ex Benth Ethanolic leaf extract on glucose transport in different cell types
Source: PeerJ. 2025 Nov 10;13:e20269. doi: 10.7717/peerj.20269 (PMC12614100; doi:10.7717/peerj.20269)
Supplement: Supplemental Information 7 [file peerj-13-20269-s007.pptx]

## Slide 1
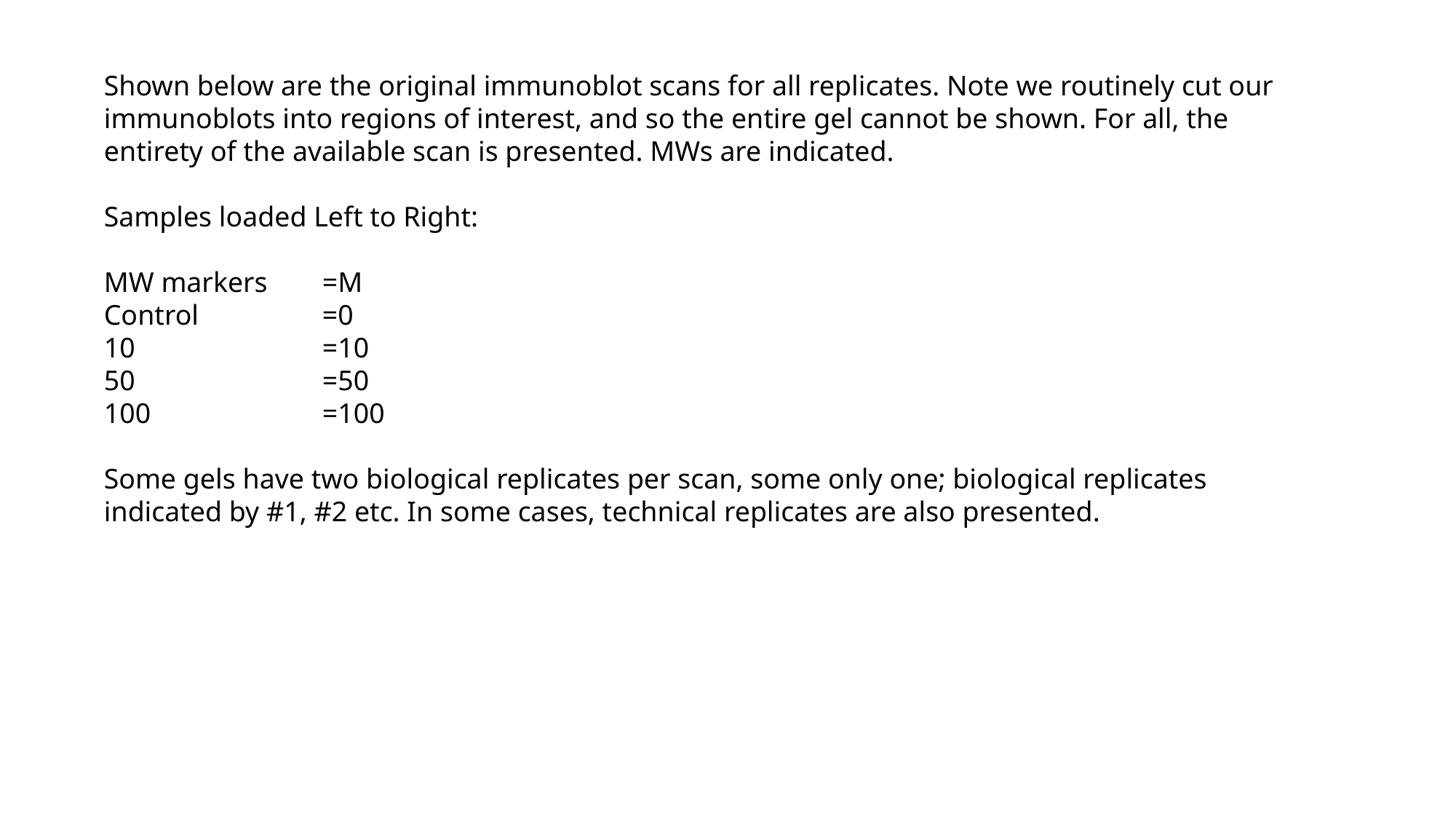

Shown below are the original immunoblot scans for all replicates. Note we routinely cut our immunoblots into regions of interest, and so the entire gel cannot be shown. For all, the entirety of the available scan is presented. MWs are indicated.
Samples loaded Left to Right:
MW markers	=M
Control		=0
10		=10
50		=50
100		=100
Some gels have two biological replicates per scan, some only one; biological replicates indicated by #1, #2 etc. In some cases, technical replicates are also presented.

## Slide 2
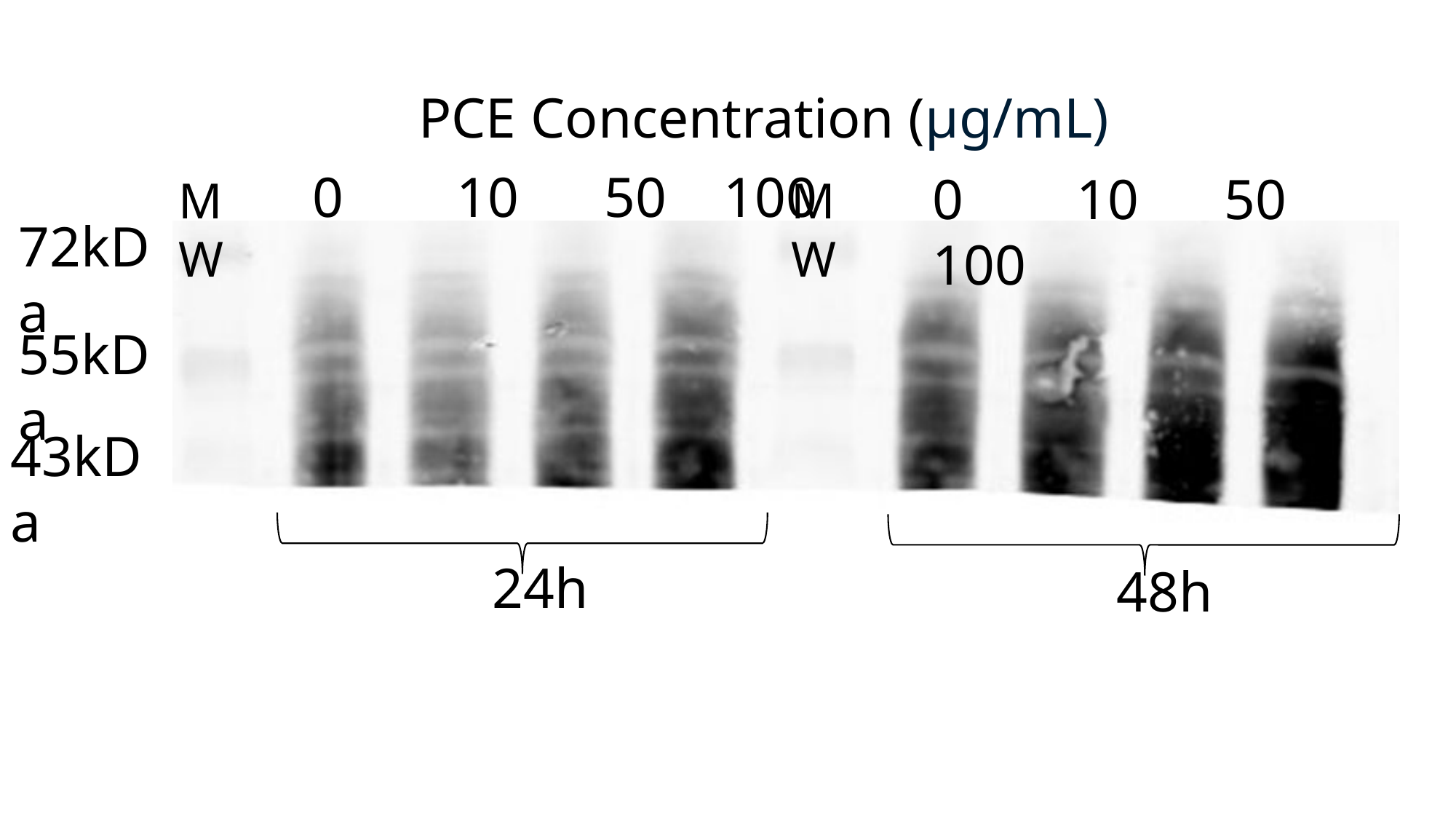

PCE Concentration (µg/mL)
0 10 50 100
0 10 50 100
MW
MW
72kDa
55kDa
43kDa
24h
48h

## Slide 3
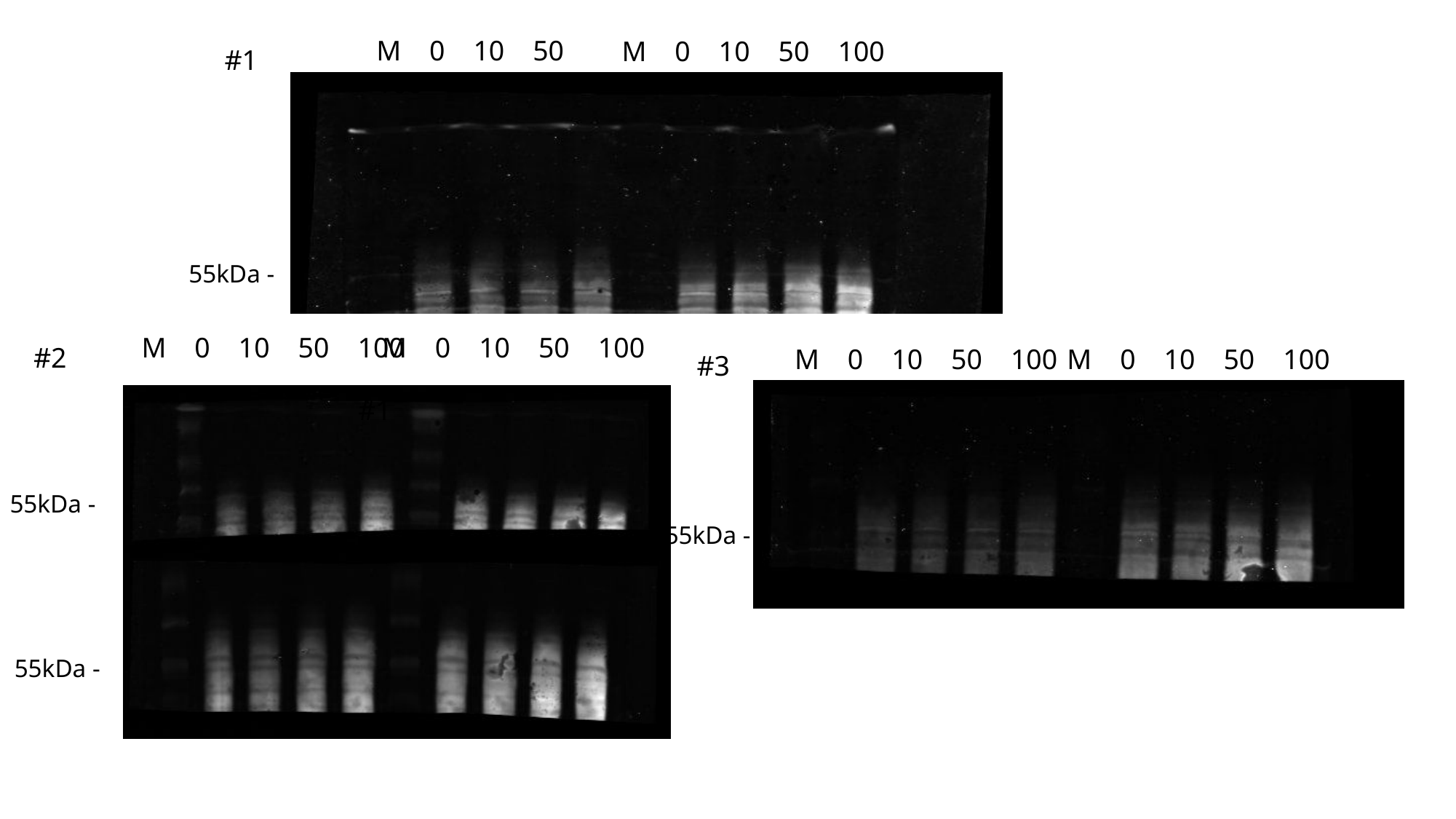

M 0 10 50 100
M 0 10 50 100
#1
55kDa -
M 0 10 50 100
M 0 10 50 100
#2
M 0 10 50 100
M 0 10 50 100
#3
#1
55kDa -
55kDa -
55kDa -

## Slide 4
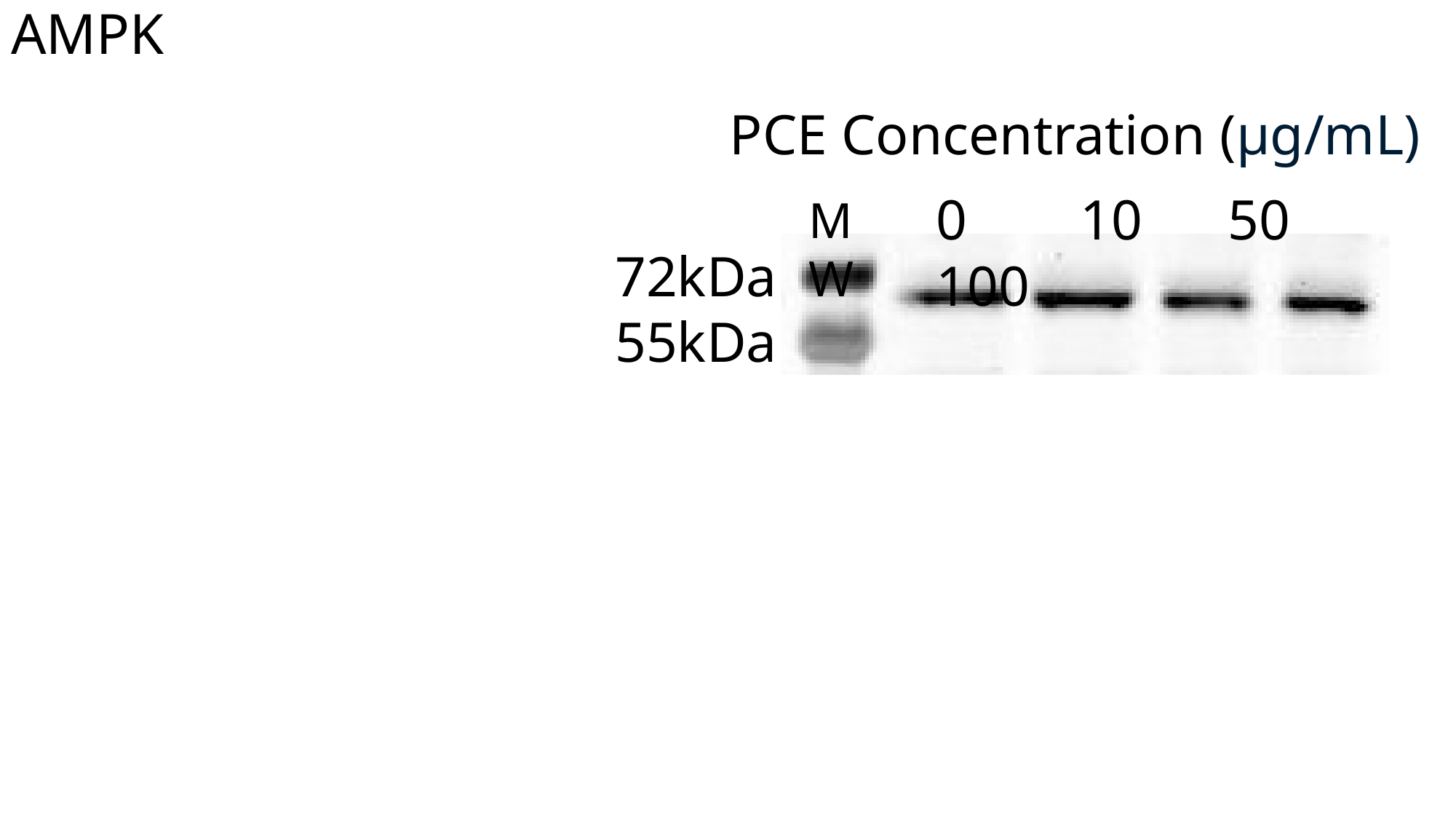

# AMPK
PCE Concentration (µg/mL)
0 10 50 100
MW
72kDa
55kDa

## Slide 5
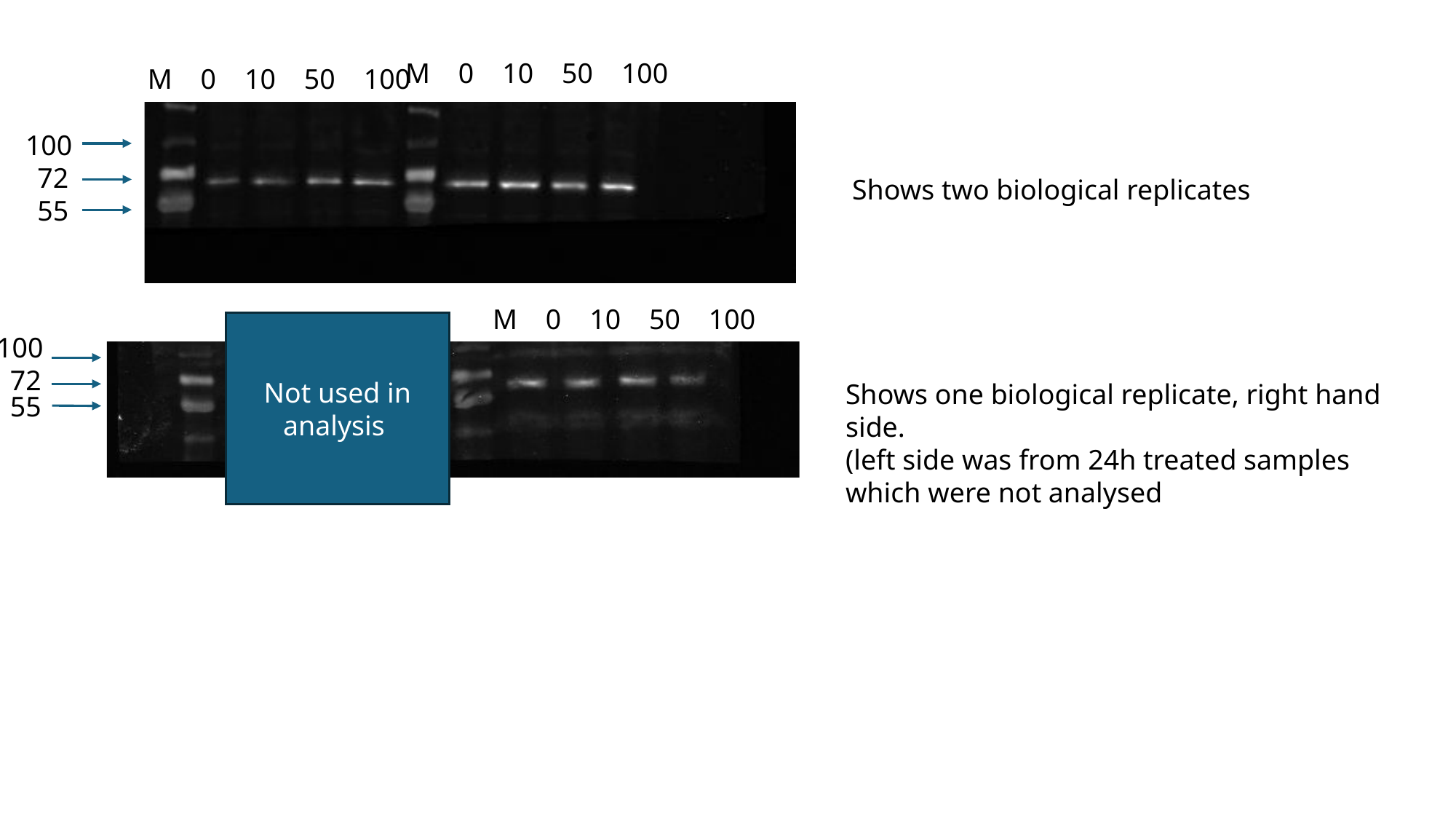

M 0 10 50 100
M 0 10 50 100
100
72
Shows two biological replicates
55
M 0 10 50 100
Not used in analysis
100
72
55
Shows one biological replicate, right hand side.
(left side was from 24h treated samples which were not analysed

## Slide 6
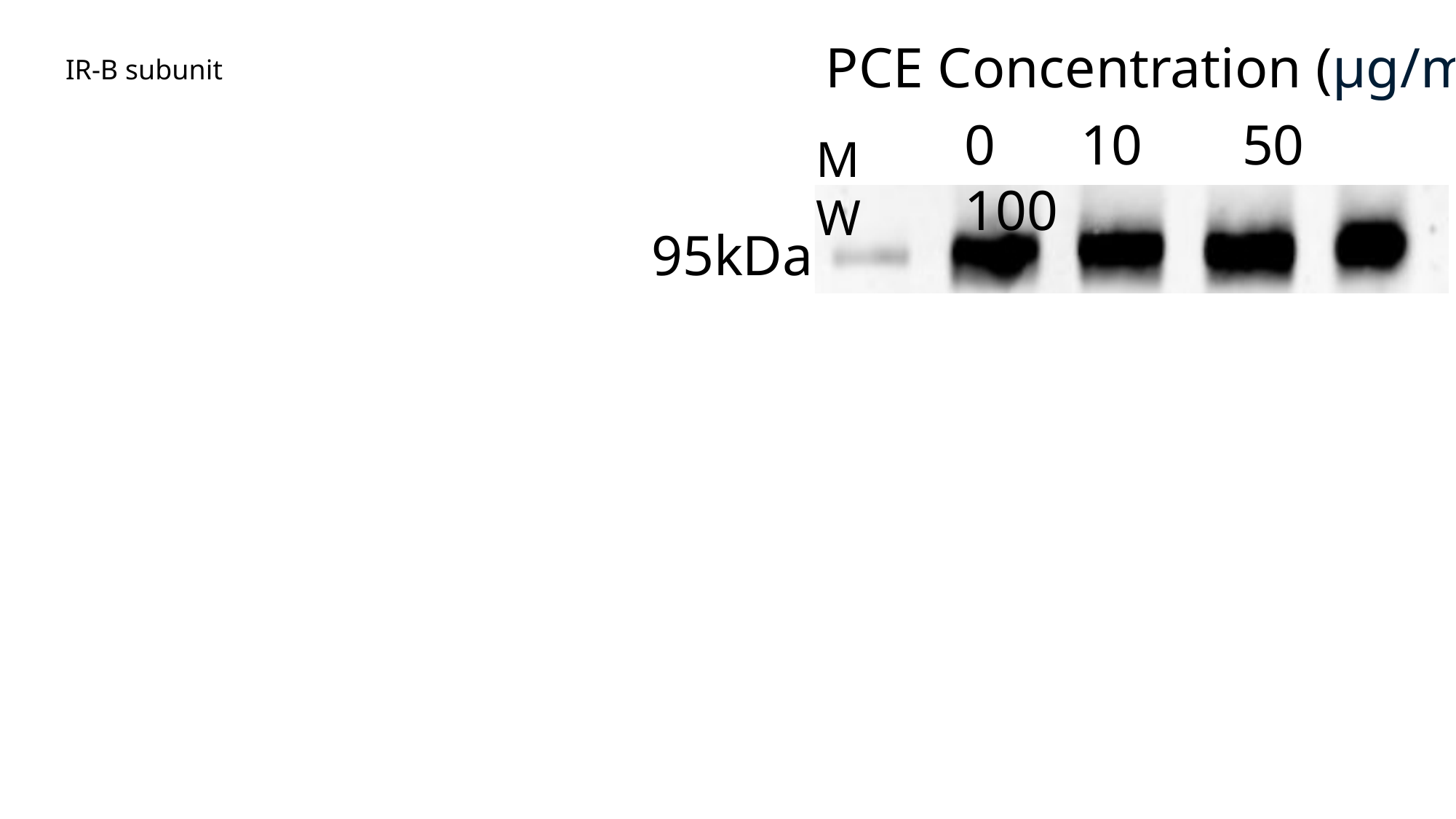

PCE Concentration (µg/mL)
IR-B subunit
0  10 50 100
MW
95kDa

## Slide 7
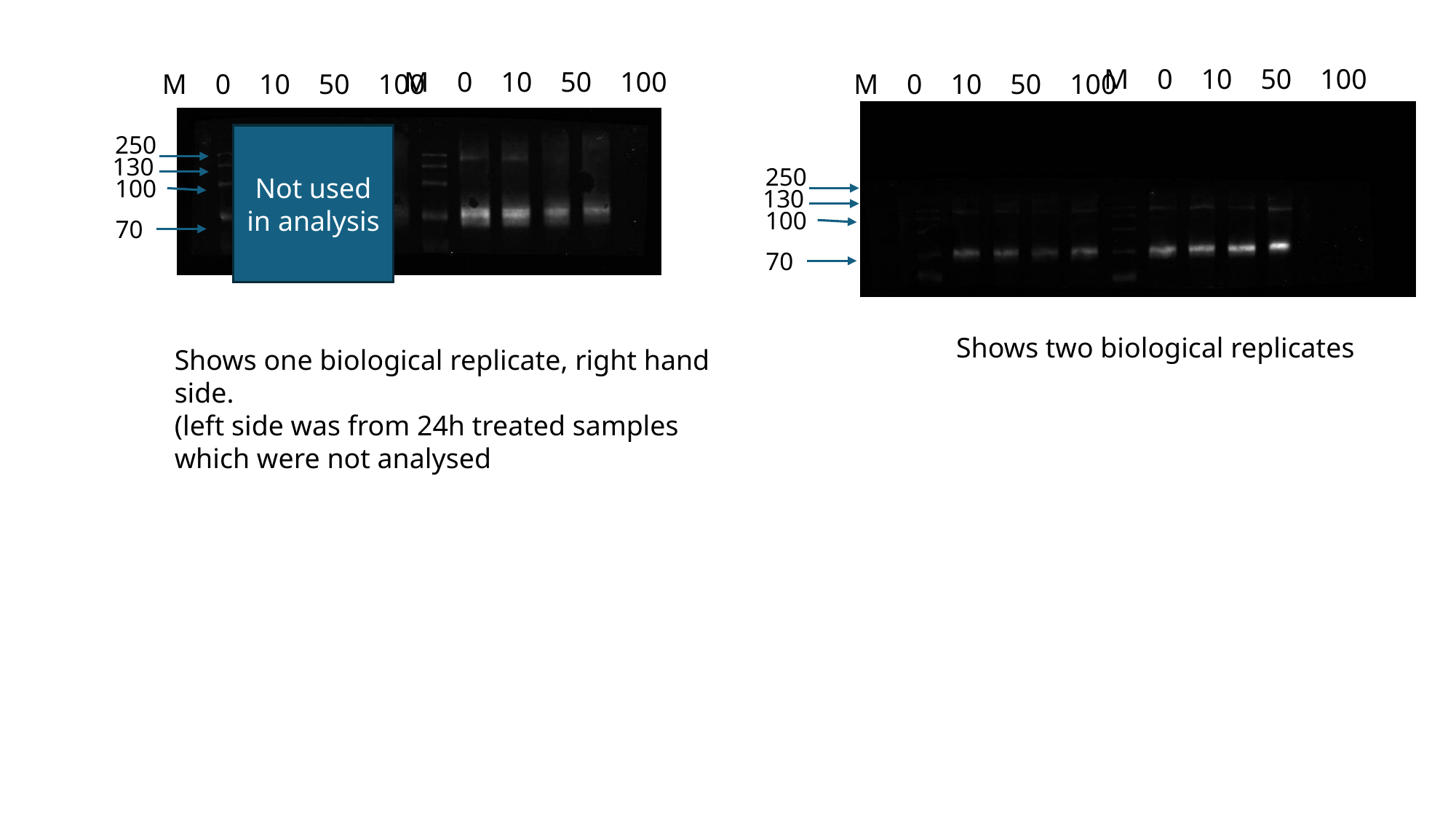

M 0 10 50 100
M 0 10 50 100
M 0 10 50 100
M 0 10 50 100
250
130
100
70
Not used in analysis
250
130
100
70
Shows two biological replicates
Shows one biological replicate, right hand side.
(left side was from 24h treated samples which were not analysed

## Slide 8
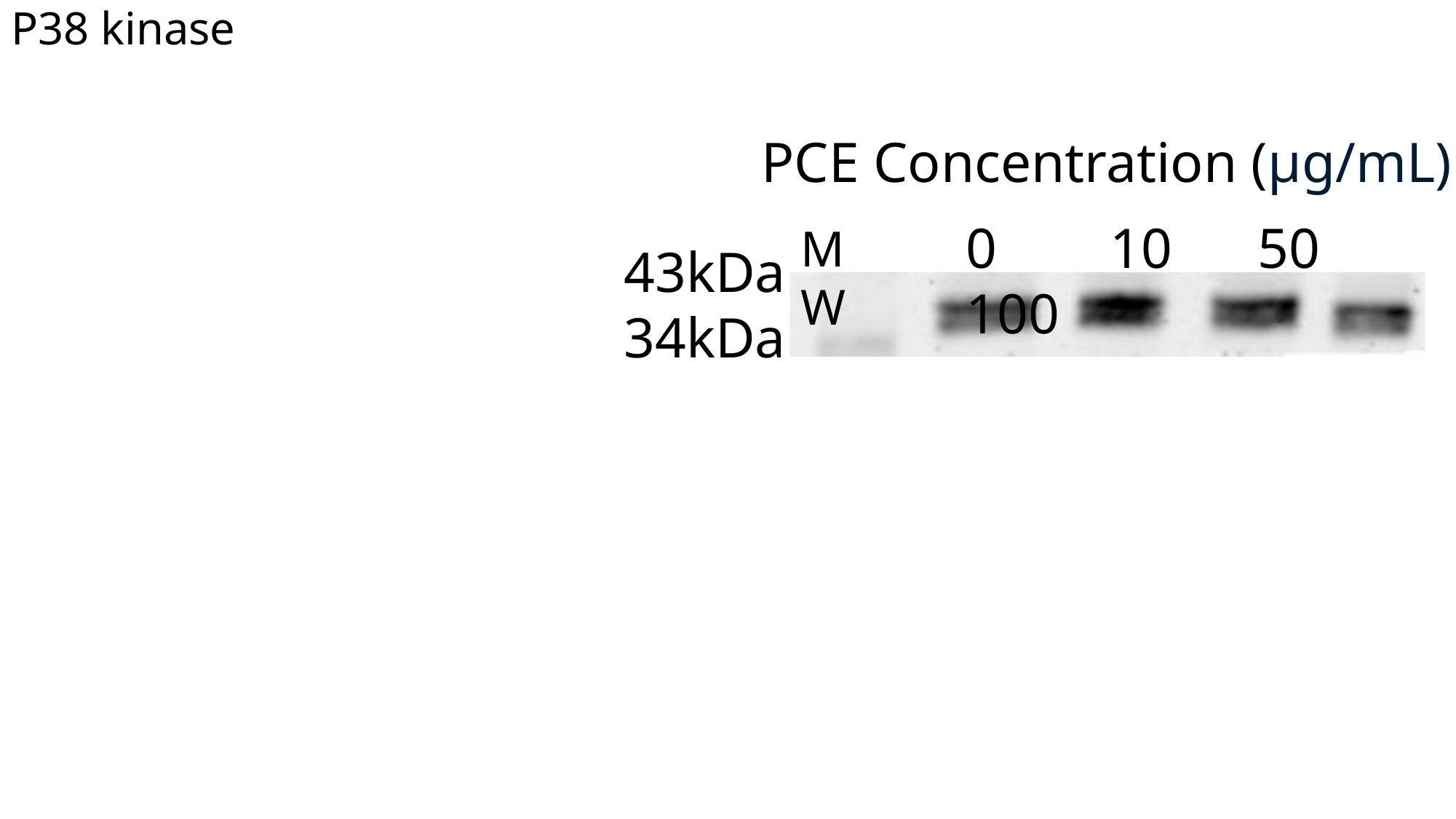

# P38 kinase
PCE Concentration (µg/mL)
0 10 50 100
MW
43kDa
34kDa

## Slide 9
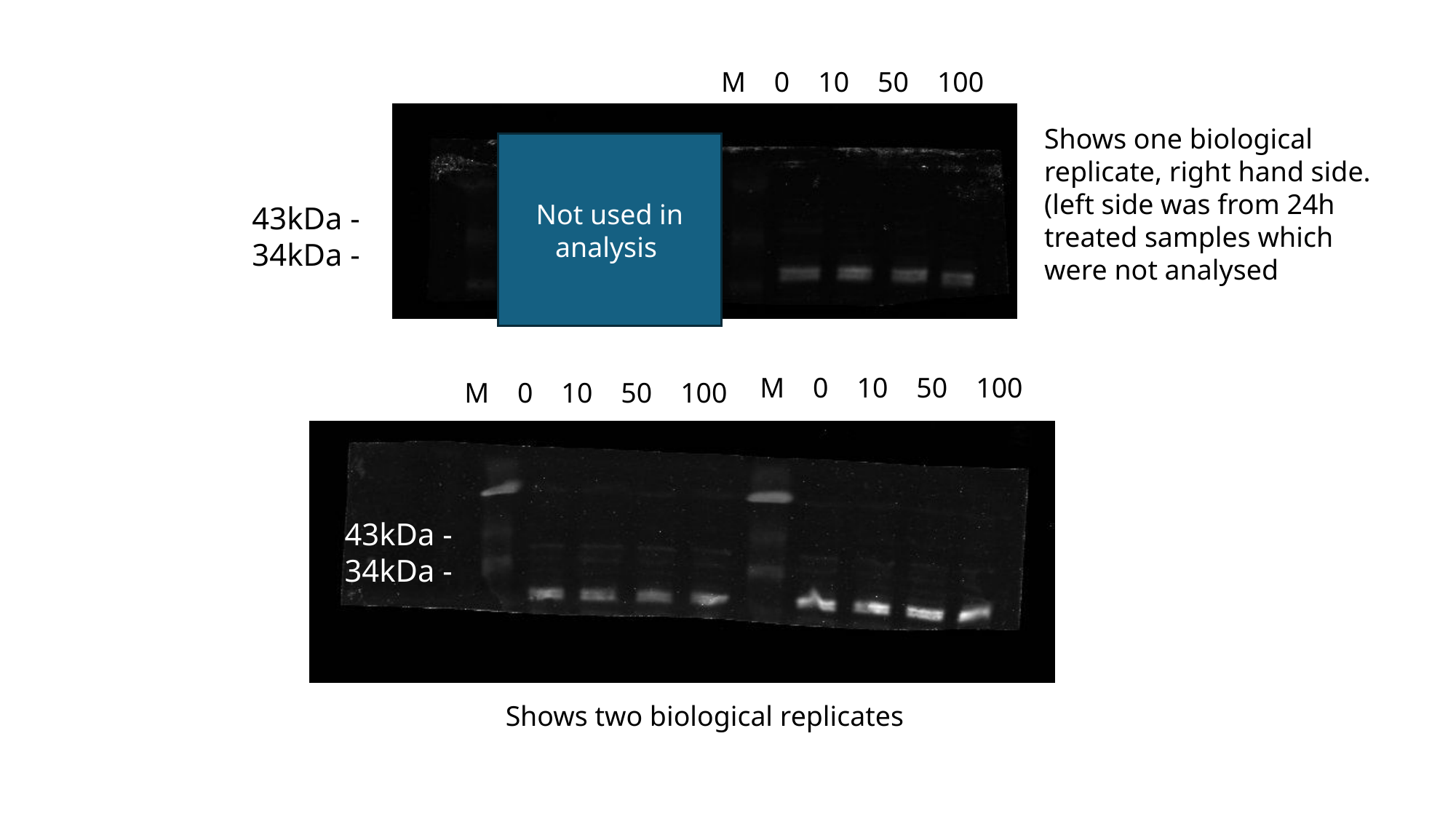

M 0 10 50 100
Shows one biological replicate, right hand side.
(left side was from 24h treated samples which were not analysed
Not used in analysis
43kDa -
34kDa -
M 0 10 50 100
M 0 10 50 100
43kDa -
34kDa -
Shows two biological replicates

## Slide 10
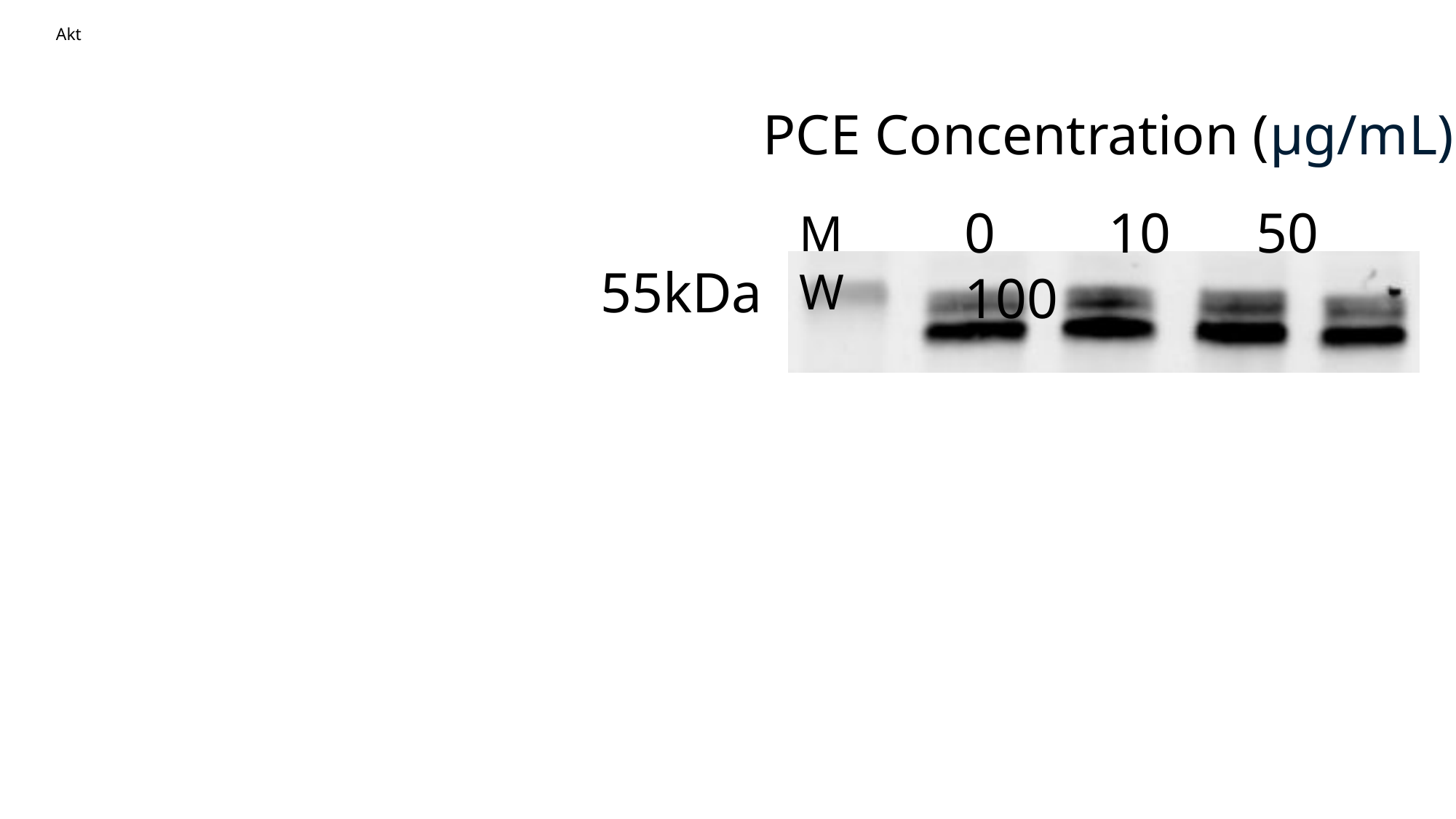

# Akt
PCE Concentration (µg/mL)
0 10 50 100
MW
55kDa

## Slide 11
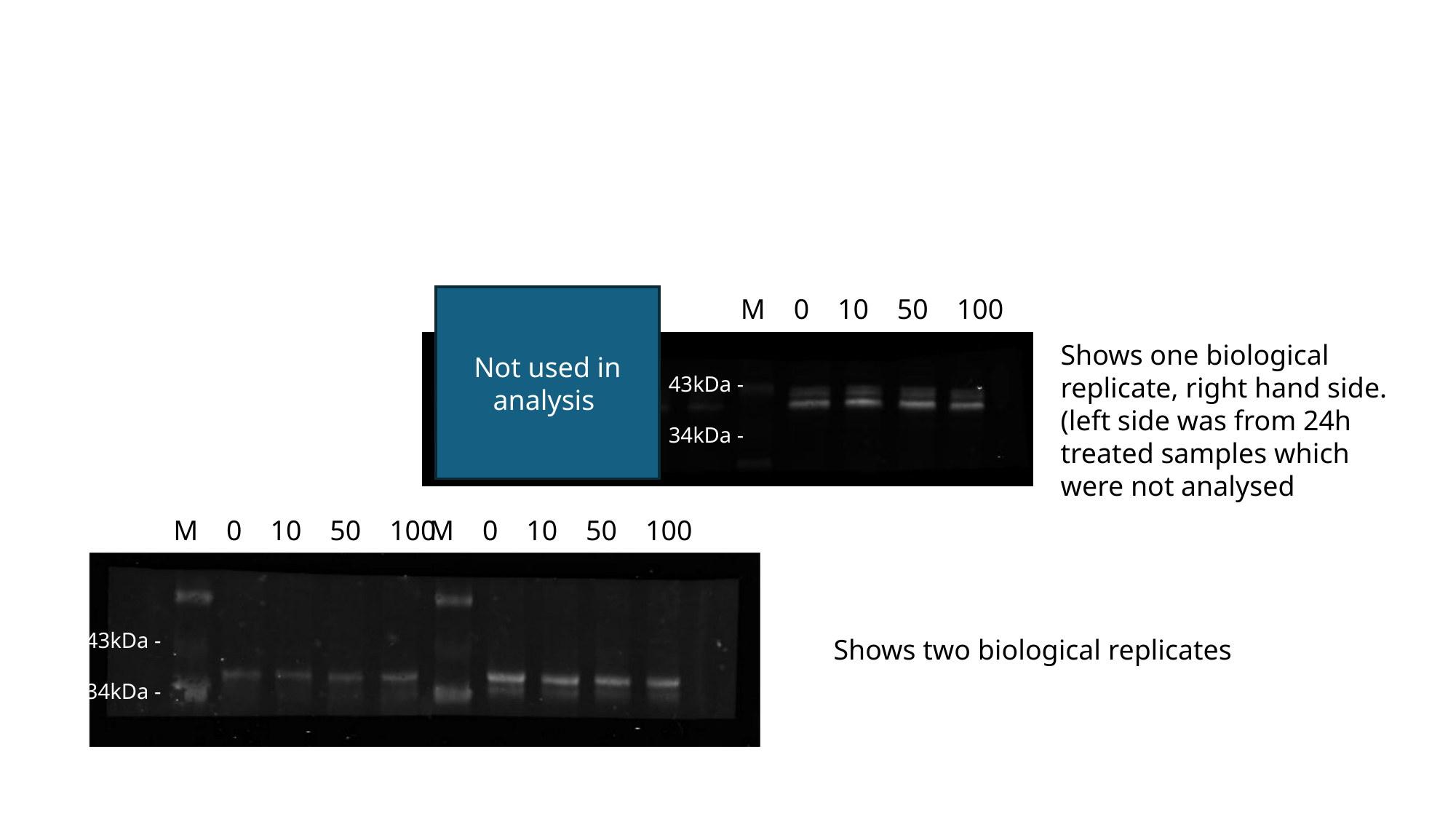

Not used in analysis
M 0 10 50 100
Shows one biological replicate, right hand side.
(left side was from 24h treated samples which were not analysed
43kDa -
34kDa -
M 0 10 50 100
M 0 10 50 100
43kDa -
34kDa -
Shows two biological replicates

## Slide 12
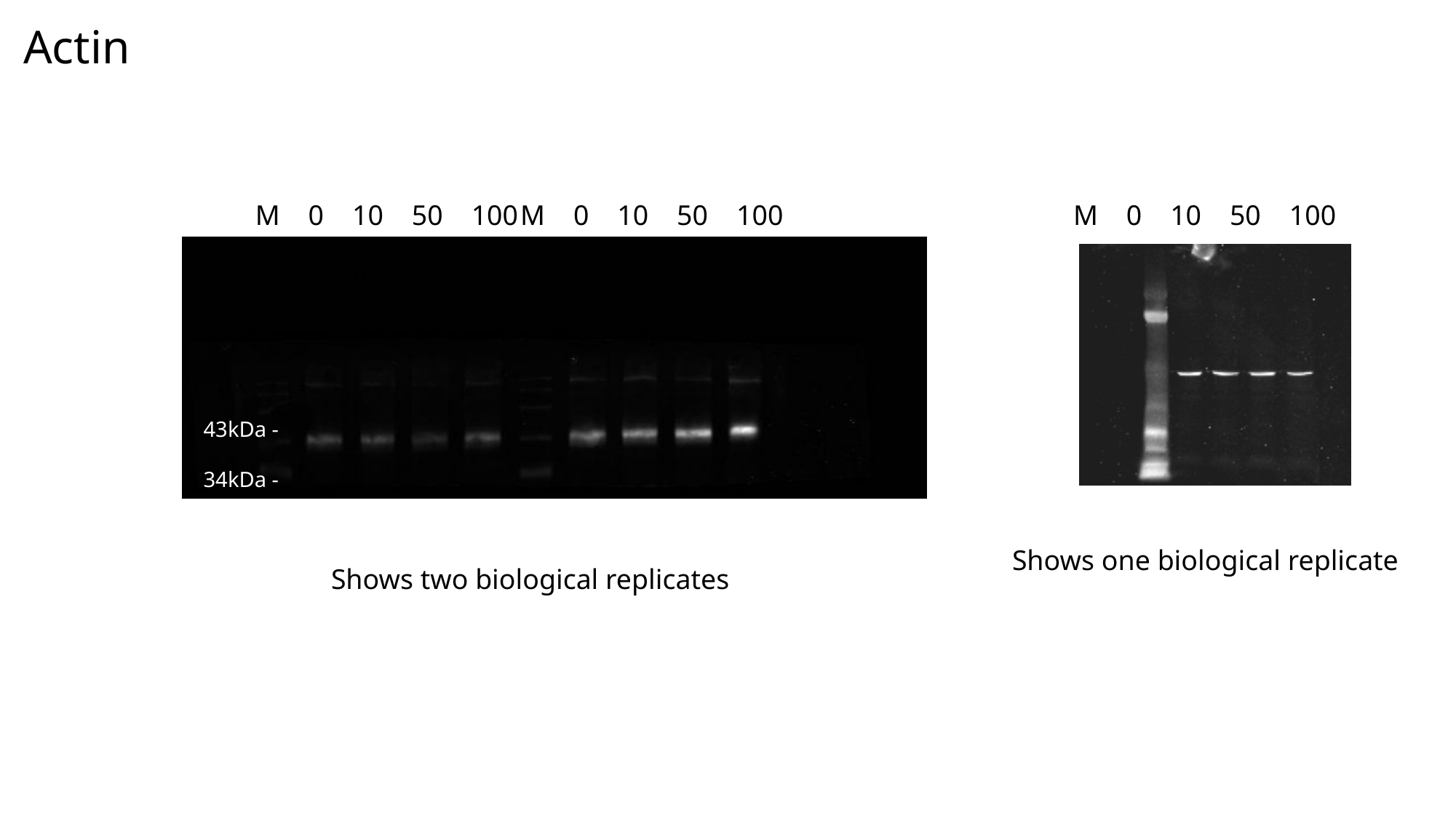

Actin
M 0 10 50 100
M 0 10 50 100
M 0 10 50 100
43kDa -
34kDa -
Shows one biological replicate
Shows two biological replicates
